# Surgical treatment options for spasticity in children and adolescents with hereditary spastic paraplegia

**DOI:** 10.1007/s00381-023-06159-w

**Published:** 2023-10-03

**Authors:** Laura A. van de Pol, Nina Burgert, Petra E. M. van Schie, K. Mariam Slot, Alida A. Gouw, Annemieke I. Buizer

**Affiliations:** 1 Department of Child Neurology, Amsterdam UMC, Vrije Universiteit, De Boelelaan 1117, Amsterdam, The Netherlands; 2 Emma Children’s Hospital, Amsterdam UMC, Meibergdreef 9, Amsterdam, The Netherlands; 3 Department of Rehabilitation Medicine, Amsterdam, UMC , Vrije Universiteit, De Boelelaan 1117, Amsterdam, The Netherlands; 4 Department of Neurosurgery, Amsterdam UMC, University of Amsterdam, Meibergdreef 9, Amsterdam, The Netherlands; 5 Department of Neurology & Clinical Neurophysiology, Amsterdam Neuroscience, Amsterdam UMC, VU University Medical Center, De Boelelaan 1117, Amsterdam, The Netherlands; 6 Rehabilitation and Development, Amsterdam Movement Sciences, Amsterdam, The Netherlands

**Keywords:** Selective dorsal rhizotomy, Intrathecal baclofen, Cerebral palsy

## Abstract

**Purpose:**

To provide an overview of outcome and complications of selective dorsal rhizotomy (SDR) and intrathecal baclofen pump implantation (ITB) for spasticity treatment in children with hereditary spastic paraplegia (HSP).

**Methods:**

Retrospective study including children with HSP and SDR or ITB. Gross motor function measure (GMFM-66) scores and level of spasticity were assessed.

**Results:**

Ten patients were included (most had mutations in *ATL1* (*n* = 4) or *SPAST* (*n* = 3) genes). Four walked without and two with walking aids, four were non-walking children. Six patients underwent SDR, three patients ITB, and one both. Mean age at surgery was 8.9 ± 4.5 years with a mean follow-up of 3.4 ± 2.2 years. Five of the SDR patients were walking. Postoperatively spasticity in the legs was reduced in all patients. The change in GMFM-66 score was + 8.0 (0–19.7 min–max). The three ITB patients treated (*SPAST* (*n* = *2*) and *PNPLA6* (*n* = *1*) gene mutation) were children with a progressive disease course. No complications of surgery occurred.

**Conclusions:**

SDR is a feasible treatment option in carefully selected children with HSP, especially in walking patients. The majority of patients benefit with respect to gross motor function, complication risk is low. ITB was used in children with severe and progressive disease.

## Introduction

Hereditary spastic paraplegia (HSP) encompasses a group of inherited neurodegenerative disorders characterized by progressive spasticity and weakness [1]. Clinically, HSP has a heterogeneous presentation and is often divided in “pure” and “complex” forms [1]. The estimated prevalence is 1.8 per 100.000 with a wide variety in age of onset [2]; a later age of onset is associated with loss of independent walking earlier in the disease course [3]. Over 80 genes have been linked to HSP so far [3]. The most frequent genotypes in a study including over 600 patients were SPG3, SPG4, SPG5, SPG7, and SPG11 [3].

No curative treatment options exist yet and current treatment is symptomatic (*for review see publication by Bellofatto*) [2]. Important goals for spasticity treatment are improving mobility and participation in daily life activities. In children, an additional important goal is prevention of muscle shortening, joint contractures, and bone deformity during growth. Besides conservative treatment options, such as physical therapy, oral spasmolytic drugs, or botulinum toxin therapy, more advanced surgical options for the long-term treatment of spasticity are available. Intrathecal baclofen (ITB) pump implantation and selective dorsal rhizotomy (SDR) have been well described as treatment options for spasticity in children [4]. Although most of the literature focusses on cerebral palsy (CP), these surgical options can also be considered in children with HSP [2, 5–9]. SDR is a single-event intervention, treating spasticity by partially transecting the posterior lumbosacral rootlets, to reduce the excitatory sensory input from the legs entering the spinal cord. The Amsterdam UMC has been performing SDR in children since 2009, as the only center in the Netherlands [10]. In CP, SDR is widely used to improve the walking pattern in ambulant children or facilitate care in non-ambulant children [11, 12]. A recent study from our center described long-term follow-up of SDR in a group of patients with a more heterogeneous etiology of spasticity, including patients with progressive neurological disorders, and showed overall gait quality improvement [10].

In contrast to CP, literature on these surgical treatment options in HSP patients is scarce. The aim of the current study is to provide an overview of outcome and complications of ITB and SDR in children and adolescents with genetically proven HSP who underwent treatment in our center. With this study, we aim to add knowledge to aid future decision-making with regard to spasticity treatment options in this specific group of patients.

### Methods

#### Study design

A retrospective study was conducted at the Amsterdam UMC. Patient files were retrospectively analyzed. The Medical Ethical Review Committee of the VU University Medical Center, Amsterdam approved the study, and consent forms were signed for all patients (reference no. 2020.407). If children were below 12 years of age, their parents signed the consent, between ages 12 and 18 years of age, both children and their parents signed the consent form.

#### Participants

All patients had a genetically confirmed diagnosis of HSP. Participants included in the study had undergone SDR or ITB, or both, between September 2009 and December 2020 in Amsterdam UMC. Children aged < 18 years at time of surgery were included in the study. Ambulant children were selected for SDR based on strict clinical criteria: bilateral spasticity that interferes with walking performance, spasticity in at least six muscle groups of the lower limbs, no severe contractures or bone deformities, sufficient strength and selective motor control, and sufficient motivation and support for the intensive postoperative rehabilitation (for detailed selection criteria see Oudenhoven et al. [10]). Non-ambulant children were selected for SDR if bilateral spasticity interfered with daily care and/or was associated with pain or discomfort. Exclusion criteria for both ambulant and non-ambulant patients for SDR were dystonia or the presence of basal ganglia involvement on brain MRI [13]. If ambulant children did not fulfill the criteria for SDR, ITB was considered to treat spasticity interfering with walking performance. If insufficient strength was the contraindication for SDR in ambulant children in level III, ITB was considered. Strength is a significant variable for both SDR and ITB treatment in GMFCS level III patients, when the goal is to improve ambulation. However, with SDR, a defined minimum level of muscle strength is necessary to guarantee preservation of walking function after loss of spasticity after surgery, because no titration is possible once nerve rootlets are cut. In some children who do not fulfill the strict SDR criteria for muscle strength, ITB can still be an option. With ITB treatment, the baclofen dose can be precisely titrated, and baclofen dosage can be adjusted (lowered) to maintain sufficient muscle tone in relatively weaker children. In non-ambulant children, treatment with ITB was an alternative for SDR to improve daily care and comfort. The choice between these two treatment options was made individually for each patient. Patients underwent standardized rehabilitation and follow-up at the department of Rehabilitation Medicine in Amsterdam UMC.

#### Study variables

The demographic variables date of birth, sex and genetic diagnosis, treatment goals and type and date of surgery, complications, age at time of surgery, and duration of follow-up were extracted from the patient files. In addition, medical records were checked for preoperative and postoperative (2 years after surgery and at time of last follow-up visit) gross motor function classification system (GMFCS) [14] levels, and for ambulant patients only, the gross motor function measure (GMFM-66) scores [15]. Although GMFCS is developed and validated for CP and cannot be used for functional prognosis in HSP, with the lack of an HSP-specific, or generic gross motor classification system, the GMFCS can be useful to describe current functional mobility in this patient group.

#### Interventions

SDR and ITB were both conducted at Amsterdam UMC. SDR was performed under general total intravenous anesthesia in prone position [13]. Patients were given antibiotic prophylaxis for 24 h postoperatively [13]. Pre-incision, subdermal electrodes were placed in standardized muscles for intraoperative mapping (bilateral m. tensor fasciae latae, m. rectus femoris, m. psoas, m. adductor brevis, m. gluteus medius, m. semimembranosus). First, en bloc laminotomy from L2 to L5 was performed. After opening the dura, dorsal roots L2 to S1 were distinguished from the ventral roots by evaluating the difference in stimulation threshold and split in mostly three (two to four) equal parts. Then, the presence/absence of sacral afferent fibers was evaluated by registering nerve action potentials in the dorsal S1-rootlets after pudendal nerve (clitoral / penile) stimulation. Rootlets containing sacral afferents were spared to avoid postoperative bladder dysfunction. Finally, each rootlet of each level was electrically stimulated with increasing intensity to assess its reflexogenic zone, indicated by both the pattern of excited compound muscle action potentials and clinical evaluation of muscle contractions by the rehabilitation physician. Those rootlets with the lowest stimulation threshold and/or most pathological reflexogenic zone were selected for rhizotomy. In most cases, the dorsal roots of L2 could not be reached for electrical stimulation and were aselectively cut for 50%. After this procedure, the laminae were re-placed and fixed with vicryl stitches or with small titanium bone plates. Patients had bed rest for the upcoming 4 days after the procedure. Mobilization conform their preoperative abilities was performed afterwards [13].

The ITB pump was implanted subfascially or in a subcutaneous pocket in the lower abdomen, depending on patient characteristics, for instance nutritional status [5]. The catheter tip was placed at level T10 if the goal was to treat spasticity of the leg muscles, and at T4 if the patient had spasticity of both legs and arms. If dystonia was prominent (in one case of complex HSP, case 6), the catheter tip was placed at cervical level (C4). To avoid infection impregnation of pump, catheter, and pocket with vancomycin solution were applied, combined with 24 h intravenous cefazolin starting 30 min before first skin incision since 2018 [16]. To prevent cerebrospinal fluid leakage, 48 h of horizontal bed rest and pressure bandage postsurgery were applied. The starting dose of intrathecal was 50 µg per 24 h for all patients. Dose elevations were assessed by the physicians [5]. To attain an optimal effect, the dose was increased in a stepwise manner by continuous delivery, except when fading of effect occurred repeatedly, then the administration was through a flexible program with periodic bolus administration [9].

## Results

All eligible patients agreed to participate in the study. Ten patients with HSP were included with a mean age of 12.8 ± 4.6 years at last follow-up, of whom six were male. Patient characteristics are shown in Table 1.

**Table 1:** Patient baseline characteristics and treatment aspects

Patient	Gene	Sex	Goal of treatment	Surgical technique	Age at surgery (years)	GMFCS score
1	*ATL1*	F	Gait improvement, less falling	SDR	5	I
2	*ATL1*	F	Gait improvement	SDR	12	I
3	*ATL1*	M	Gait improvement and contracture prevention	SDR	3	III
4	*ATL1*	M	Comfort/care giving/contracture prevention	SDR	6	IV
5	*SPAST*	F	Gait improvement	SDR	4	III
6	*SPAST*	M	See case description	SDR and ITB	10 (SDR), 5 and 12 (ITB)	III
7	*SPAST*	M	Care	ITB	7	IV
8	*SPAST*	M	Care and positioning in wheelchair	ITB	12	IV
9	*KIF1A*	F	Gait improvement	SDR	17	I
10	*PNPLA6*	M	Painful gait	ITB	10	III

*F* female, *M* male, *SDR* selective dorsal rhizotomy, *ITB* intrathecal baclofen pump, *GMFCS* gross motor function classification system

SDR was performed in six patients, ITB in three patients, and one patient underwent both. Four different genetic subtypes of HSP were found, with mutations in the ATL*1* (*n* = 4), *SPAST* (*n* = 4), *KIF1A* (*n* = 1), *PNPLA6* (*n* = 1) genes. Mean age at surgery, for the nine patients who underwent one procedure only, was 8.9 ± 4.5 years with a mean follow-up of 3.4 ± 2.2 years. No postoperative complications occurred, apart from temporary postoperative urinary retention in one ITB patient.

Table 2 presents the pre- and postoperative characteristics of the patients who underwent solely SDR. GMFCS scores did not change after surgery. Four patients remained stable, patient 2 showed clear improvement in motor function. In four patients, spasticity was no longer present after surgery; in two patients, low-level spasticity remained only in the soleus muscle (uni- or bilaterally). In all patients, treatment goals were met.

**Table 2:** Pre- and postoperative characteristics of the SDR patients

Patient	Interval operation date and last follow-up (years)	GMFM-66 score pre: 2 year post: last follow-up	Spasticity at follow-up
1	1.1	80.93:NA:80.90	-
2	2.9	86.52:NA:100.00	-
3	3.0	53.86:55.39:55.39	SOL bilateral
4	5.1	22.66:NA:NA	-
5	3.1	47.68:53.38:53.09	-
9	3.0	100.00:100.00:100.00	SOL right

Follow-up scores. GMFM-66 scores were not complete for all patients. In patient 1, surgery had taken place less than 2 years ago. Patient 2 missed the 2-year follow-up because of logistic reasons, and patient 4 was too severely impaired to use the GMFM-66 score for follow-up measurements

*pre* preoperative, *post* postoperative, *NA* not available, *GMFM* gross motor function measure, *SDR* selective dorsal rhizotomy *SOL* soleus muscle

Table 3 shows the percentage of selected rootlets during SDR in the five ambulant.

**Table 3:** Percentages of transected rootles per level patients

Patient		Sacral afferents S1	Total	S1 (%)	L5 (%)	L4 (%)	L3 (%)	L2 (%)
1	left	yes	37	45	33	33	40	33
	right	yes	37	40	33	40	40	33
2	left	no	41	50	40	33	33	50 (aselective)
	right	no	39	50	30	33	33	50 (aselective)
3	left	no	36	33	30	33	33	50 (aselective)
	right	no	40	40	33	30	45	50 (aselective)
5	left	no	38	33	40	33	33	50 (aselective)
	right	no	36	33	33	33	33	50 (aselective)
9	left	no	37	33	50	33	33	NA
	right	no	37	50	33	33	33	NA

Numbers are percentages of transected rootlets per level

*NA* not applicable, *S* sacral, *L* lumbal

Below, we describe the cases of four patients that were treated with ITB.

Patient 6, a boy with HSP based on a de novo mutation in the *SPAST* gene, functioned at a level comparable to GMFCS level III at age five. Caretaking and walking were disturbed by the high level of spasticity. As he had insufficient strength for SDR, an ITB pump was implanted with the tip on level T10, with the goal to improve caretaking and aided walking. With progressive disease, he was no longer able to walk or to sit independently; a high level of spasticity in the legs caused problems in care and positioning in the wheelchair despite ITB. At the age of 10 years, the ITB pump was removed, and an SDR was performed to reduce leg spasticity. Two years later, he developed anarthria, swallowing difficulties and severe generalized dystonia of trunk and back, with additional posturing of upper extremities, with a low level of spasticity, again interfering with sitting and positioning in the wheelchair. To reduce dystonia, an ITB pump was reimplanted, with the catheter tip at level C4, with a positive effect on muscle tone and comfort that lasted until the last visit at the age of 16 years.

Patient 7, a boy with HSP based on a de novo mutation in the *SPAST* gene, functioned at a level comparable to GMFCS level IV at age 7; the high level of leg spasticity made caretaking difficult. An ITB pump was implanted with the catheter tip at level Th10. This resulted in a satisfying reduction of muscle tone and improvement of caretaking that lasted until the last follow-up visit at the age of 15 years. Over the years, he developed a progressive dysarthria and scoliosis.

Patient 8, a boy with HSP based on a de novo mutation in the *SPAST* gene, functioned at a level comparable to GMFCS level IV, at age 12. The high level of leg spasticity combined with muscle contractures made positioning in his wheelchair and caretaking difficult. An ITB pump was recently implanted with the catheter tip at level Th10. At one-month follow-up, the reduced spasticity in his legs has clearly improved sitting and caregiving.

Patient 10 is a boy with HSP based on two heterozygous mutations in the *PNPLA6* gene. He presented with bilateral spasticity, functioning at a level comparable to GMFCS level III, causing discomfort and knee pain during walking. Because he had insufficient muscle strength to fulfill the criteria for SDR, an ITB pump was implanted at age ten, to improve comfort, which had a positive effect on comfort during walking and transfers. With progression of the disease, the peripheral motor neuron component became more apparent with increasing hypotonia of the extremities and loss of tendon reflexes. The dosage of the ITB pump was gradually decreased until, at the age of 17 years, the ITB pump was removed.

## Discussion

In this study, we described children with genetically proven HSP who underwent surgical spasticity treatment. This is the largest study to date describing children with a genetically proven type of HSP treated with SDR and/or ITB.

Regarding SDR, all six patients from our center had reduced spasticity in the lower extremities postoperatively. At follow-up, ranging from 1.1 to 5.1 years, the reduction of spasticity was maintained, while motor function remained stable or improved. Although SDR is an established treatment in children with spastic CP [8, 11, 12], very limited data are available on SDR in children with genetically proven HSP. In a recent review that addressed SDR in spasticity of genetic origin, only seven children with HSP were identified, aged between 3 and 10 years at time of surgery [17]. In only four of these children, an underlying genetic diagnosis was described, of whom two patients were from our center, and included in the current study as well [13]. The other two patients, described in the literature with a known genetic diagnosis, were two siblings with *ALS2* gene-related HSP who had a GMFCS level of III and IV and were aged 3 and 7 years, respectively at time of SDR [18]. Both patients had a postoperative improvement in muscle tone but a functional decline related to the severe and progressive subtype of HSP. Another three children diagnosed with HSP, without clear genetic diagnosis, were aged 6 or 7 years at time of SDR had GMFCS levels of I and II and a follow-up ranging from 2 to 14 years. Of these three children, two improved regarding gross motor function and one deteriorated [18, 19].

Another complicating factor, when studying the outcome of SDR in HSP, is the underlying genetic heterogeneity. Below age ten, *ATL1-*related HSP (SPG3A) is a frequent cause of HSP. Regarding prognosis, the progression rate in this subgroup of HSP is generally slow, and wheelchair dependency or need for a walking aid (cane, walker, or wheelchair) is relatively rare [20]. In our current study, there were four children with a mutation in the *ATL1* gene, of whom one was described earlier [13]. Of the four children in our study, disease severity varied from walking independently to being wheelchair dependent. All remained stable after SDR, while they benefitted from the reduction in spasticity regarding their treatment goals. However, follow-up was relatively short varying between 1.1 and 5.1 years. We found no other studies describing outcome of SDR in patients with *ATL1-*related HSP. The other two children with SDR in our study had mutations in the *SPAST* and *KIFA1* gene. SDR in these genetic HSP subtypes has not previously been described in children, nor in adults. Despite the small number of patients, our study suggests that there is a growing experience that carefully selected children with HSP can benefit from SDR. Since clinical progression in HSP, even within genetic subtypes, may be variable, it is important to take the rate of progression in each individual patient into account, especially when indicating SDR, an irreversible procedure.

We reported on four, non-ambulatory, children with HSP treated with ITB of whom three had a de novo mutation in the *SPAST* gene. In two patients, ITB improved caretaking and/or positioning in the wheelchair; in two other patients, further treatment was necessary. In the literature, only six children with HSP and ITB treatment have been described, in whom only one had a known genetic diagnosis, and the majority were walking children, which makes it difficult to compare our experience with more severely affected HSP patients with known genetic diagnosis [21–23]. Pucks-Faes et al. described retrospectively seven patients with HSP who were treated with ITB, one of whom was a child, aged between 10 and 14 years. This child had a sporadic complex form of HSP, without reported genetic diagnosis. Treatment with ITB resulted in an improvement of clinical and functional parameters after follow-up of 3 years. In the total group of included patients in this study, of whom in only one patient the genetic diagnosis was reported (a *SPAST* gene mutation). Pucks-Faes et al. noted three stages after ITB implantation; improvement of spasticity during 2–3 years, followed by a stable phase of 4–5 years. Thereafter, the maintenance or progressive loss of mobility depended on the individual disease course [24]. Recently, Pointon et al. described five children with HSP, of whom four were without a genetic diagnosis and one with a mutation in the *KIF1A* gene, who were treated with ITB. They reported that ITB treatment in these walking patients (aged 5–10 years) was associated with a reduction in spasticity and a trend toward improvement in quality of life and patient centered goals [22]. Coulter et al. identified 11 studies assessing ITB treatment in 58 patients (57 adults and one child) with HSP, including the study by Pucks-Faes described above [21]. Of these patients, genetic diagnosis was reported in only two patients (a *SPAST* and a *SPG7* gene mutation). They conclude that most patients with ITB treatment seem to benefit from it, although they suggest there may be a possible selection bias if only patients who responded to a test dose were implanted. They also report that not all patients are responsive, possibly explained by the heterogeneity in underlying mutations [21].

Strengths of our study are that all patients had a genetically confirmed diagnoses of HSP, and that clinical follow-up, in the SDR patients, was performed following a standardized procedure. A drawback of this study is the relatively short follow-up. Another drawback is the relatively small group of patients; however, in comparison to previous papers, this is the largest case-series on children and adolescents with genetically proven HSP that underwent surgical spasticity treatment.

## Conclusions

We conclude that SDR and ITB are safe and feasible treatment options in children and adolescents with HSP. The advantage of SDR is that it is a single procedure. A possible disadvantage may be that it is irreversible, although in our patients no negative effects were present. Whereas ITB treatment has the disadvantage of more hospital visits for pump refilling and long-term complications, such as catheter dysfunction, the advantage of ITB is that the baclofen dosage is adjustable and treatment is reversible, by removing the pump if needed. The indication for surgical spasticity treatment and the choice for SDR or ITB must be made carefully for the individual child or adolescent with HSP, depending on level of functioning, treatment goals, comorbidity, and estimation of the prognosis of the underlying genetic subtype. In relative stable forms of HSP SDR is an option in well selected children and adolescents who fulfill the criteria for SDR. Because of the rarity and genetic heterogeneity of HSP, it is very important to report the underlying genetic diagnosis, perform long-term follow-up, and use standardized outcome measures in future studies to be able to combine international data on this subject.

## Data Availability

Not applicable.
